# 2,3-Diphenyl-2,3-di­hydro-4*H*-pyrido[3,2-*e*][1,3]thia­zin-4-one

**DOI:** 10.1107/S1600536814009714

**Published:** 2014-05-03

**Authors:** Hemant P. Yennawar, Harnoor Singh, Lee J. Silverberg

**Affiliations:** a Department of Chemistry, Pennsylvania State University, University Park, PA 16802, USA; b Pennsylvania State University, Schuylkill Campus, 200 University Drive, Schuylkill Haven, PA 17972, USA

## Abstract

In the racemic title compound, C_19_H_14_N_2_OS, the two phenyl substituents on the 1,3-thia­zine ring are almost perpendicular to the pyridine ring which is fused to the thia­zine ring [inter-ring dihedral angles = 87.90 (8) and 85.54 (7)°]. The dihedral angle between the two phenyl rings is 75.11 (7)°. The six-membered thia­zine ring has an envelope conformation with the *ortho*-related C atom forming the flap. The crystals exhibit face-to-edge aromatic-ring interactions with the nearest C—H⋯C distance equal to 3.676 (3) Å.

## Related literature   

For the syntheses and crystal structures of related compounds, see: Yennawar *et al.* (2013 ▶, 2014 ▶); Yennawar & Silverberg (2013 ▶, 2014 ▶). For the formation of amide bonds using 2,4,6-tripropyl-1,3,5,2,4,6-trioxatri­phospho­rinane-2,4,6-trioxide (T3P) and pyridine, see: Dunetz *et al.* (2011 ▶). For the microwave-promoted reaction of an *N*-aryl imine with 2-thio­nicotinic acid, see: Dandia *et al.* (2004 ▶).
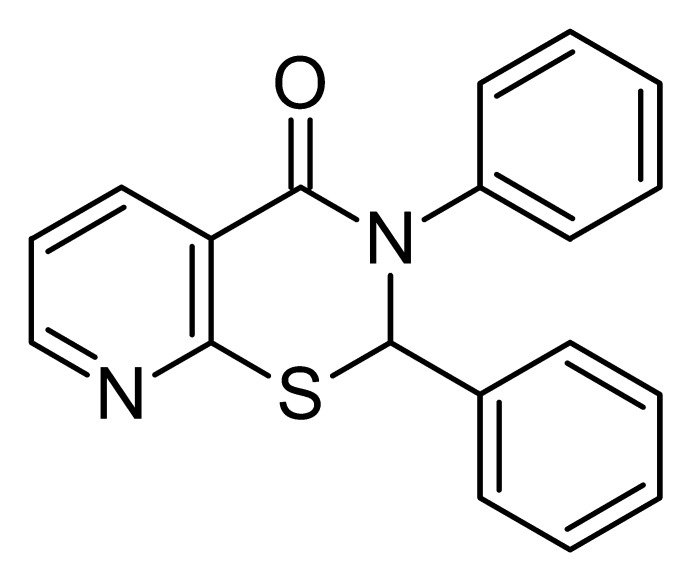


## Experimental   

### 

#### Crystal data  

C_19_H_14_N_2_OS
*M* *_r_* = 318.38
Triclinic, 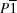

*a* = 9.069 (7) Å
*b* = 9.772 (7) Å
*c* = 10.150 (7) Å
α = 80.320 (11)°
β = 63.737 (10)°
γ = 78.591 (12)°
*V* = 787.4 (10) Å^3^
*Z* = 2
Mo *K*α radiation
μ = 0.21 mm^−1^
*T* = 298 K
0.29 × 0.23 × 0.20 mm

#### Data collection  

Bruker SMART APEX CCD diffractometer
Absorption correction: multi-scan (*SADABS*; Sheldrick, 2004 ▶) *T*_min_ = 0.941, *T*_max_ = 0.959
7363 measured reflections
3795 independent reflections
3322 reflections with *I* > 2σ(*I*)
*R*_int_ = 0.013

#### Refinement  

*R*[*F* ^2^ > 2σ(*F* ^2^)] = 0.041
*wR*(*F* ^2^) = 0.114
*S* = 1.05
3795 reflections
208 parameters
H-atom parameters not refined
Δρ_max_ = 0.34 e Å^−3^
Δρ_min_ = −0.28 e Å^−3^

### 

Data collection: *SMART* (Bruker, 2001 ▶); cell refinement: *SAINT* (Bruker, 2001 ▶); data reduction: *SAINT*; program(s) used to solve structure: *SHELXS97* (Sheldrick, 2008 ▶); program(s) used to refine structure: *SHELXL97* (Sheldrick, 2008 ▶); molecular graphics: *XSHELL* (Bruker, 2001 ▶); software used to prepare material for publication: *ORTEP-3 for Windows* (Farrugia, 2012 ▶).

## Supplementary Material

Crystal structure: contains datablock(s) I. DOI: 10.1107/S1600536814009714/zs2297sup1.cif

Structure factors: contains datablock(s) I. DOI: 10.1107/S1600536814009714/zs2297Isup2.hkl

Supporting information file. DOI: 10.1107/S1600536814009714/zs2297Isup3.mol

Supporting information file. DOI: 10.1107/S1600536814009714/zs2297Isup4.cml

CCDC reference: 1000248

Additional supporting information:  crystallographic information; 3D view; checkCIF report

## Figures and Tables

**Fig. 1.**
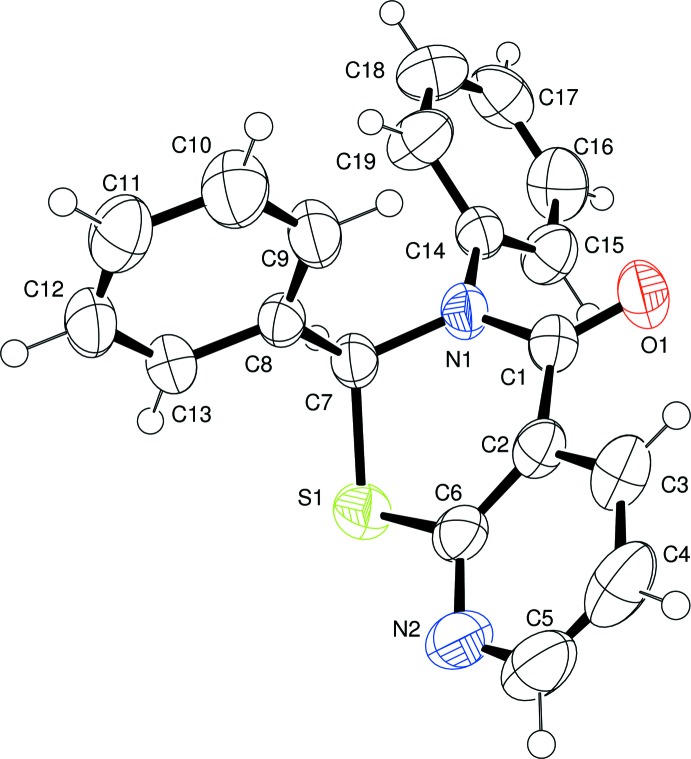
An ORTEP view of the title comound. Thermal ellipsoids are drawn at the 50% probability level.

**Fig. 2.**
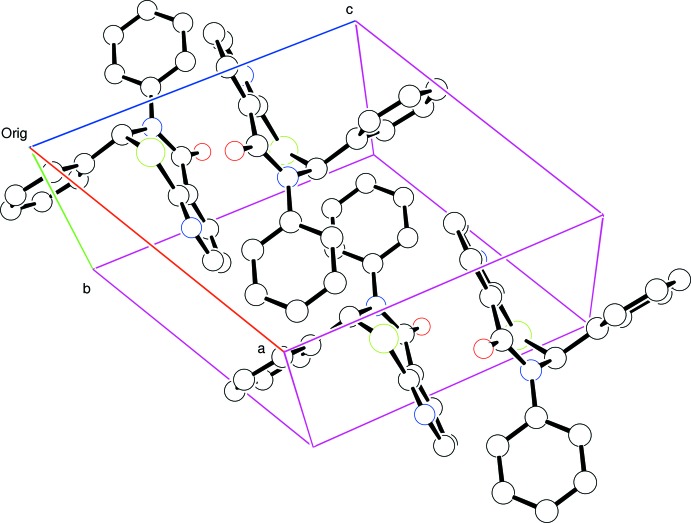
Crystal packing in the unit cell.

## supplementary crystallographic information

### 1. Comment

Dandia *et al.* (2004) have reported that in the attempted reaction of *N*-(4-methylphenyl)-1-phenylmethanimine with 2-thionicotinic acid at 142 °C for 26 h, no product formed, which they attributed to the "low reactivity" of 2-thionicotinic acid. However, under microwave irradiation for 10 minutes in DMF, the reaction gave an 85% yield of the desired 3-(4-methylphenyl)-2-phenyl-2,3-dihydro-4*H*-pyrido[3,2-*e*][1,3]thiazin-4-one. This appears to be the only previous report of a 2,3-diaryl-2,3-dihydro-4*H*-pyrido[3,2-*e*][1,3]thiazin-4-one. We report here the synthesis of 2,3-diphenyl-2,3-dihydro-4*H*-pyrido[3,2-*e*][1,3]thiazin-4-one, the title compound, at room temperature, without the use of microwaves, by the reaction of *N*-benzylideneaniline with 2-thionicotinic acid using 2,4,6-tripropyl-1,3,5,2,4,6-trioxatriphosphorinane-2,4,6-trioxide (T3P) and pyridine (Dunetz *et al.*, 2011; Yennawar & Silverberg, 2013, 2014; Yennawar *et al.*, 2013, 2014). This compound continues our study of the structures of 1,3-thiaza-4-one heterocycles (Yennawar *et al.*, 2013, 2014; Yennawar & Silverberg, 2013, 2014), the most closely analogous compound among them being 2,3-diphenyl-2,3-dihydro-4*H*-1,3-benzothiazin-4-one (Yennawar *et al.*, 2014), which has a benzene ring fused to the 1,3-thiazin-4-one ring instead of the pyridine ring, as reported here.

In the racemic title compound, C_19_H_14_N_2_OS (Fig. 1), the two phenyl substituents on the 1,3-thiazine ring are almost perpendicular to the pyridine ring which is fused to the thiazine ring [pyridyl to benzene inter-ring dihedral angles are 87.90 (8) and 85.54 (7)°]. The dihedral angle between the two benzene rings is 75.11 (7)°. The six-membered thiazine ring has an envelope conformation with the *ortho*-related carbon (C7) forming the flap. In the crystal, no formal intermolecular hydrogen bonds are present but face-to-edge interactions between the aromatic rings are found (Fig. 2).

### 2. Experimental

A two-necked 25 ml round bottom flask was oven-dried, cooled under N_2_, and charged with a stirring bar and *N*-benzylideneaniline (1.087 g, 6 mmol). Tetrahydrofuran (2.3 ml) was added, the solid dissolved, and the solution was stirred. Pyridine (1.95 ml, 24 mmol) and then 2-thionicotinic acid (0.931 g, 6 mmol) were added. Finally, 2,4,6-tripropyl-1,3,5,2,4,6-trioxatriphosphorinane-2,4,6-trioxide in 2-methyltetrahydrofuran (50 weight percent; 7.1 ml, 12 mmol) was added. The reaction was stirred at room temperature until completed as indicated by TLC, then poured into a separatory funnel along with dichloromethane and water. The layers were separated and the aqueous was extracted twice with dichloromethane. The organics were combined and washed with saturated sodium bicarbonate and saturated sodium chloride. The organic layer was dried over sodium sulfate, concentrated under vacuum and chromatographed on 30 g flash silica gel, eluting with mixtures of ethyl acetate and hexanes (10% to 50% ethyl acetate). The product was eluted with 40–50% EtOAc/hexanes and was concentrated under vacuum to give a solid (0.8724 g, 45.7%). Recrystallization from ethanol gave a white solid (0.6927 g, 36.3%). m.p. 134–135 °C; *R*_f_ = 0.33 (40% EtOAc/hexanes). Crystals for X-ray crystallography were grown by slow evaporation from ethanol.

### 3. Refinement

The C-bound H atoms were geometrically placed with C—H = 0.93–0.97 Å, and refined as riding with *U*_iso_(H) = 1.2*U*_eq_(C).

### Crystal data

**Table:**

C_19_H_14_N_2_OS	*Z* = 2
*M**_r_* = 318.38	*F*(000) = 332
Triclinic, *P*1	*D*_x_ = 1.343 Mg m^−^^3^
Hall symbol: -P 1	Melting point: 407.5 K
*a* = 9.069 (7) Å	Mo *K*α radiation, λ = 0.71073 Å
*b* = 9.772 (7) Å	Cell parameters from 4119 reflections
*c* = 10.150 (7) Å	θ = 2.3–28.2°
α = 80.320 (11)°	µ = 0.21 mm^−^^1^
β = 63.737 (10)°	*T* = 298 K
γ = 78.591 (12)°	Block, colourless
*V* = 787.4 (10) Å^3^	0.29 × 0.23 × 0.20 mm

### Data collection

**Table:**

Bruker SMART APEX CCD diffractometer	3795 independent reflections
Radiation source: fine-focus sealed tube	3322 reflections with *I* > 2σ(*I*)
Graphite monochromator	*R*_int_ = 0.013
Detector resolution: 8.34 pixels mm^-1^	θ_max_ = 28.3°, θ_min_ = 2.1°
φ and ω scans	*h* = −12→12
Absorption correction: multi-scan (*SADABS*; Sheldrick, 2004)	*k* = −12→11
*T*_min_ = 0.941, *T*_max_ = 0.959	*l* = −13→13
7363 measured reflections	

### Refinement

**Table:**

Refinement on *F*^2^	Primary atom site location: structure-invariant direct methods
Least-squares matrix: full	Secondary atom site location: difference Fourier map
*R*[*F*^2^ > 2σ(*F*^2^)] = 0.041	Hydrogen site location: inferred from neighbouring sites
*wR*(*F*^2^) = 0.114	H-atom parameters not refined
*S* = 1.05	*w* = 1/[σ^2^(*F*_o_^2^) + (0.0559*P*)^2^ + 0.1486*P*] where *P* = (*F*_o_^2^ + 2*F*_c_^2^)/3
3795 reflections	(Δ/σ)_max_ = 0.002
208 parameters	Δρ_max_ = 0.34 e Å^−^^3^
0 restraints	Δρ_min_ = −0.28 e Å^−^^3^

### Special details

**Table:**

Experimental. Absorption correction: SADABS (Sheldrick, 2004) was used for absorption correction. *R*_int_ was 0.0331 before and 0.0128 after correction. The ratio of minimum to maximum transmission is 0.8482. The λ/2 correction factor is 0.0015.The data collection nominally covered a full sphere of reciprocal space by a combination of 4 sets of ω scans each set at different φ and/or 2θ angles and each scan (10 s exposure) covering -0.300° degrees in ω. The crystal to detector distance was 5.82 cm.
Geometry. All e.s.d.'s (except the e.s.d. in the dihedral angle between two l.s. planes) are estimated using the full covariance matrix. The cell e.s.d.'s are taken into account individually in the estimation of e.s.d.'s in distances, angles and torsion angles; correlations between e.s.d.'s in cell parameters are only used when they are defined by crystal symmetry. An approximate (isotropic) treatment of cell e.s.d.'s is used for estimating e.s.d.'s involving l.s. planes.
Refinement. Refinement of *F*^2^ against ALL reflections. The weighted *R*-factor *wR* and goodness of fit *S* are based on *F*^2^, conventional *R*-factors *R* are based on *F*, with *F* set to zero for negative *F*^2^. The threshold expression of *F*^2^ > σ(*F*^2^) is used only for calculating *R*-factors(gt) *etc*. and is not relevant to the choice of reflections for refinement. *R*-factors based on *F*^2^ are statistically about twice as large as those based on *F*, and *R*- factors based on ALL data will be even larger.

### Fractional atomic coordinates and isotropic or equivalent isotropic displacement parameters (Å^2^)

**Table:**

	*x*	*y*	*z*	*U*_iso_*/*U*_eq_	
C1	0.19222 (17)	0.19834 (14)	0.51794 (14)	0.0451 (3)	
C2	0.01727 (17)	0.24186 (15)	0.62281 (14)	0.0467 (3)	
C3	−0.1009 (2)	0.1554 (2)	0.65325 (17)	0.0602 (4)	
H3	−0.0686	0.0659	0.6213	0.072*	
C4	−0.2667 (2)	0.2042 (3)	0.7316 (2)	0.0766 (6)	
H4	−0.3480	0.1474	0.7561	0.092*	
C5	−0.3088 (2)	0.3386 (3)	0.7723 (2)	0.0799 (6)	
H5	−0.4211	0.3726	0.8197	0.096*	
C6	−0.03959 (18)	0.37357 (16)	0.67724 (15)	0.0498 (3)	
C7	0.27666 (16)	0.35802 (14)	0.62977 (14)	0.0430 (3)	
H7	0.3722	0.4092	0.5913	0.052*	
C8	0.26430 (15)	0.28466 (13)	0.77829 (13)	0.0412 (3)	
C9	0.2928 (2)	0.14162 (16)	0.80549 (17)	0.0562 (4)	
H9	0.3126	0.0841	0.7334	0.067*	
C10	0.2920 (3)	0.08234 (19)	0.9406 (2)	0.0701 (5)	
H10	0.3106	−0.0146	0.9582	0.084*	
C11	0.2641 (2)	0.1654 (2)	1.04781 (18)	0.0660 (4)	
H11	0.2652	0.1252	1.1372	0.079*	
C12	0.2347 (2)	0.3081 (2)	1.02200 (17)	0.0620 (4)	
H12	0.2153	0.3650	1.0944	0.074*	
C13	0.23370 (18)	0.36766 (16)	0.88894 (16)	0.0526 (3)	
H13	0.2123	0.4646	0.8730	0.063*	
C14	0.47645 (16)	0.24896 (14)	0.40084 (14)	0.0435 (3)	
C15	0.4999 (2)	0.29060 (18)	0.25726 (16)	0.0576 (4)	
H15	0.4095	0.3254	0.2351	0.069*	
C16	0.6598 (2)	0.2800 (2)	0.14615 (18)	0.0723 (5)	
H16	0.6762	0.3074	0.0489	0.087*	
C17	0.7934 (2)	0.23006 (19)	0.1770 (2)	0.0689 (5)	
H17	0.9002	0.2233	0.1013	0.083*	
C18	0.7697 (2)	0.1902 (2)	0.3191 (2)	0.0736 (5)	
H18	0.8609	0.1576	0.3406	0.088*	
C19	0.6115 (2)	0.1978 (2)	0.43170 (19)	0.0666 (4)	
H19	0.5963	0.1684	0.5283	0.080*	
N1	0.31171 (14)	0.26429 (12)	0.51779 (12)	0.0452 (3)	
N2	−0.19949 (17)	0.42352 (18)	0.74849 (15)	0.0673 (4)	
O1	0.22485 (14)	0.11017 (12)	0.43317 (12)	0.0589 (3)	
S1	0.09809 (5)	0.49046 (4)	0.64714 (4)	0.05549 (13)	

### Atomic displacement parameters (Å^2^)

**Table:**

	*U*^11^	*U*^22^	*U*^33^	*U*^12^	*U*^13^	*U*^23^
C1	0.0498 (7)	0.0485 (7)	0.0426 (6)	−0.0085 (5)	−0.0234 (6)	−0.0060 (5)
C2	0.0463 (7)	0.0579 (8)	0.0415 (6)	−0.0094 (6)	−0.0234 (5)	−0.0029 (5)
C3	0.0595 (9)	0.0792 (11)	0.0528 (8)	−0.0234 (8)	−0.0304 (7)	0.0010 (7)
C4	0.0540 (9)	0.1285 (18)	0.0571 (9)	−0.0349 (10)	−0.0282 (8)	0.0058 (10)
C5	0.0447 (9)	0.1375 (19)	0.0563 (9)	−0.0019 (10)	−0.0224 (7)	−0.0157 (11)
C6	0.0488 (7)	0.0625 (8)	0.0417 (6)	−0.0003 (6)	−0.0251 (6)	−0.0056 (6)
C7	0.0435 (6)	0.0461 (7)	0.0435 (6)	−0.0081 (5)	−0.0189 (5)	−0.0109 (5)
C8	0.0369 (6)	0.0487 (7)	0.0423 (6)	−0.0065 (5)	−0.0177 (5)	−0.0118 (5)
C9	0.0744 (10)	0.0499 (8)	0.0532 (8)	−0.0098 (7)	−0.0323 (7)	−0.0109 (6)
C10	0.0976 (13)	0.0576 (9)	0.0649 (10)	−0.0148 (9)	−0.0447 (10)	0.0030 (8)
C11	0.0705 (10)	0.0854 (12)	0.0470 (8)	−0.0160 (9)	−0.0290 (7)	−0.0011 (8)
C12	0.0624 (9)	0.0820 (11)	0.0492 (8)	−0.0037 (8)	−0.0266 (7)	−0.0246 (8)
C13	0.0561 (8)	0.0541 (8)	0.0553 (8)	−0.0011 (6)	−0.0282 (7)	−0.0200 (6)
C14	0.0448 (7)	0.0458 (6)	0.0415 (6)	−0.0055 (5)	−0.0183 (5)	−0.0090 (5)
C15	0.0584 (9)	0.0695 (9)	0.0472 (7)	−0.0113 (7)	−0.0256 (7)	0.0002 (7)
C16	0.0765 (11)	0.0902 (13)	0.0435 (8)	−0.0266 (10)	−0.0146 (8)	−0.0020 (8)
C17	0.0527 (9)	0.0664 (10)	0.0707 (11)	−0.0141 (7)	−0.0042 (8)	−0.0190 (8)
C18	0.0475 (8)	0.0818 (12)	0.0854 (13)	0.0007 (8)	−0.0265 (8)	−0.0082 (10)
C19	0.0529 (9)	0.0902 (12)	0.0555 (9)	−0.0032 (8)	−0.0271 (7)	0.0001 (8)
N1	0.0431 (6)	0.0556 (6)	0.0402 (5)	−0.0071 (5)	−0.0172 (4)	−0.0136 (5)
N2	0.0490 (7)	0.0977 (11)	0.0548 (7)	0.0103 (7)	−0.0262 (6)	−0.0176 (7)
O1	0.0654 (6)	0.0604 (6)	0.0578 (6)	−0.0134 (5)	−0.0254 (5)	−0.0196 (5)
S1	0.0646 (2)	0.0454 (2)	0.0607 (2)	0.00138 (16)	−0.03260 (19)	−0.00882 (15)

### Geometric parameters (Å, º)

**Table:**

C1—O1	1.2220 (17)	C9—H9	0.9300
C1—N1	1.3653 (18)	C10—C11	1.371 (3)
C1—C2	1.491 (2)	C10—H10	0.9300
C2—C3	1.391 (2)	C11—C12	1.369 (3)
C2—C6	1.397 (2)	C11—H11	0.9300
C3—C4	1.381 (3)	C12—C13	1.381 (2)
C3—H3	0.9300	C12—H12	0.9300
C4—C5	1.373 (3)	C13—H13	0.9300
C4—H4	0.9300	C14—C19	1.376 (2)
C5—N2	1.333 (3)	C14—C15	1.377 (2)
C5—H5	0.9300	C14—N1	1.4371 (18)
C6—N2	1.332 (2)	C15—C16	1.385 (2)
C6—S1	1.7511 (18)	C15—H15	0.9300
C7—N1	1.4654 (17)	C16—C17	1.362 (3)
C7—C8	1.522 (2)	C16—H16	0.9300
C7—S1	1.8230 (17)	C17—C18	1.359 (3)
C7—H7	0.9800	C17—H17	0.9300
C8—C9	1.374 (2)	C18—C19	1.380 (3)
C8—C13	1.3921 (19)	C18—H18	0.9300
C9—C10	1.393 (2)	C19—H19	0.9300
			
O1—C1—N1	122.08 (13)	C12—C11—C10	119.39 (15)
O1—C1—C2	120.66 (12)	C12—C11—H11	120.3
N1—C1—C2	117.23 (12)	C10—C11—H11	120.3
C3—C2—C6	117.43 (14)	C11—C12—C13	120.27 (14)
C3—C2—C1	118.63 (14)	C11—C12—H12	119.9
C6—C2—C1	123.37 (13)	C13—C12—H12	119.9
C4—C3—C2	119.06 (18)	C12—C13—C8	120.93 (15)
C4—C3—H3	120.5	C12—C13—H13	119.5
C2—C3—H3	120.5	C8—C13—H13	119.5
C5—C4—C3	118.40 (17)	C19—C14—C15	119.68 (14)
C5—C4—H4	120.8	C19—C14—N1	120.54 (13)
C3—C4—H4	120.8	C15—C14—N1	119.74 (13)
N2—C5—C4	124.37 (17)	C14—C15—C16	119.18 (15)
N2—C5—H5	117.8	C14—C15—H15	120.4
C4—C5—H5	117.8	C16—C15—H15	120.4
N2—C6—C2	123.93 (15)	C17—C16—C15	121.06 (17)
N2—C6—S1	114.61 (13)	C17—C16—H16	119.5
C2—C6—S1	121.38 (12)	C15—C16—H16	119.5
N1—C7—C8	115.03 (12)	C18—C17—C16	119.54 (16)
N1—C7—S1	111.09 (9)	C18—C17—H17	120.2
C8—C7—S1	112.49 (9)	C16—C17—H17	120.2
N1—C7—H7	105.8	C17—C18—C19	120.59 (17)
C8—C7—H7	105.8	C17—C18—H18	119.7
S1—C7—H7	105.8	C19—C18—H18	119.7
C9—C8—C13	118.39 (13)	C14—C19—C18	119.94 (16)
C9—C8—C7	123.51 (11)	C14—C19—H19	120.0
C13—C8—C7	117.95 (13)	C18—C19—H19	120.0
C8—C9—C10	120.29 (13)	C1—N1—C14	120.09 (11)
C8—C9—H9	119.9	C1—N1—C7	122.27 (11)
C10—C9—H9	119.9	C14—N1—C7	117.56 (11)
C11—C10—C9	120.73 (17)	C5—N2—C6	116.65 (17)
C11—C10—H10	119.6	C6—S1—C7	96.48 (9)
C9—C10—H10	119.6		
			
O1—C1—C2—C3	18.1 (2)	C14—C15—C16—C17	−0.4 (3)
N1—C1—C2—C3	−164.00 (12)	C15—C16—C17—C18	−0.1 (3)
O1—C1—C2—C6	−152.95 (14)	C16—C17—C18—C19	1.1 (3)
N1—C1—C2—C6	24.91 (19)	C15—C14—C19—C18	0.8 (3)
C6—C2—C3—C4	1.5 (2)	N1—C14—C19—C18	−176.76 (16)
C1—C2—C3—C4	−170.08 (13)	C17—C18—C19—C14	−1.4 (3)
C2—C3—C4—C5	2.1 (2)	O1—C1—N1—C14	10.5 (2)
C3—C4—C5—N2	−3.9 (3)	C2—C1—N1—C14	−167.36 (11)
C3—C2—C6—N2	−4.0 (2)	O1—C1—N1—C7	−172.88 (13)
C1—C2—C6—N2	167.17 (13)	C2—C1—N1—C7	9.29 (18)
C3—C2—C6—S1	179.39 (10)	C19—C14—N1—C1	−122.82 (16)
C1—C2—C6—S1	−9.42 (18)	C15—C14—N1—C1	59.58 (19)
N1—C7—C8—C9	3.60 (18)	C19—C14—N1—C7	60.37 (19)
S1—C7—C8—C9	132.15 (13)	C15—C14—N1—C7	−117.22 (15)
N1—C7—C8—C13	179.04 (11)	C8—C7—N1—C1	77.79 (15)
S1—C7—C8—C13	−52.41 (14)	S1—C7—N1—C1	−51.45 (16)
C13—C8—C9—C10	−0.5 (2)	C8—C7—N1—C14	−105.48 (13)
C7—C8—C9—C10	174.93 (15)	S1—C7—N1—C14	125.27 (11)
C8—C9—C10—C11	−0.5 (3)	C4—C5—N2—C6	1.6 (3)
C9—C10—C11—C12	0.8 (3)	C2—C6—N2—C5	2.5 (2)
C10—C11—C12—C13	−0.2 (3)	S1—C6—N2—C5	179.27 (12)
C11—C12—C13—C8	−0.8 (2)	N2—C6—S1—C7	156.14 (11)
C9—C8—C13—C12	1.1 (2)	C2—C6—S1—C7	−26.97 (12)
C7—C8—C13—C12	−174.57 (13)	N1—C7—S1—C6	53.86 (10)
C19—C14—C15—C16	0.1 (2)	C8—C7—S1—C6	−76.72 (10)
N1—C14—C15—C16	177.69 (14)		
